# Draft Genome Sequences of *Bifidobacterium* Strains N4G05 and N5G01, Isolated from the Human Vaginal Microbiome

**DOI:** 10.1128/genomeA.01433-17

**Published:** 2018-01-11

**Authors:** Aline C. Freitas, Janet E. Hill

**Affiliations:** a Department of Veterinary Microbiology, University of Saskatchewan, Saskatoon, Saskatchewan, Canada

## Abstract

We report here the draft genome sequences of *Bifidobacterium* strains N4G05 and N5G01, isolated from the human vaginal microbiome. Genome sequences were obtained by *de novo* assembly from high-quality reads. Both strains were closely related to *Bifidobacterium kashiwanohense* based on barcode marker sequences and average nucleotide identity analysis.

## GENOME ANNOUNCEMENT

*Bifidobacterium* spp. are Gram-positive, anaerobic, nonmotile, non-spore-forming, rod-shaped bacteria. They colonize the human gut, vagina, oral cavity, breast milk, and environmental sources (1–3). Bifidobacteria play an important role in the human gut microbiome due their health-promoting characteristics, such as immune modulation (4, 5) and inhibition of pathogens (6–9). They contribute to vaginal homeostasis by producing lactic acid and can be the dominant organisms in vaginal microbiomes of healthy reproductive-age women (10, 11). Here, we present the draft genome sequences of *Bifidobacterium* strains N4G05 and N5G01, isolated from the human vaginal microbiome.

Genomic DNA was isolated from cultures grown in modified reinforced clostridial broth by a modified salting-out procedure (12). Libraries were prepared using the Nextera XT DNA library preparation kit, according to the manufacturer’s instructions (Illumina, Inc., San Diego, CA). PhiX DNA (15% [vol/vol]) was added to the indexed libraries prior to loading onto the flow cell. The libraries were sequenced using the reagent kit version 2 (500 cycles) on the Illumina MiSeq platform (Illumina, Inc.). Raw sequence reads were trimmed using Trimmomatic (13), with a minimum read length of 40 bp and quality cutoff Phred score of 20. High-quality reads were *de novo* assembled with Geneious Assembler (Geneious version 11.0). The genomes were annotated using the National Center for Biotechnology Information Prokaryotic Genome Annotation Pipeline. The average nucleotide identity by MUMmer (ANIm) was calculated within JSpecies (14).

*Bifidobacterium* N4G05 was assembled into 12 contigs, with a total sequence length of 2.07 Mb and a GC content of 56.1%. Average coverage across genome was 27×. The *N*_50_ and *N*_90_ values were 352,734 and 95,710 bp, respectively. The bacterial barcodes cpn60 (15) and rpoB (16) indicated that N4G05 was closely related to *Bifidobacterium kashiwanohense*, with an identity of 98% (*cpn60*, 552 bp) and 99% (*rpoB*, 3,561 bp). The sequence of the 16S rRNA gene (1,538 bp) had a similarity of 98% to *B. kashiwanohense* and 99% to both *Bifidobacterium catenulatum* and *Bifidobacterium pseudocatenulatum*. The genomic ANIm of N4G05 was on the borderline of the suggested cutoff for species identity (95 to 96% [17]), with a maximum of ANIm of 95.2% with *B. kashiwanohense*.

*Bifidobacterium* N5G01 was assembled into 8 contigs with a total sequence length of 2.12 Mb and a GC content of 56.2%. Average coverage across the genome was 26×. *N*_50_ and *N*_90_ values were 515,170 and 134,235, respectively. The comparison with cpn60 and rpoB suggested that N5G01 was closely related to *B. kashiwanohense*, with an identity of 98% (cpn60, 552 bp) and 99% (rpoB, 3560 bp), while barcode 16S rRNA (1538 bp) had a similarity of 99% to *B. kashiwanohense*, *B. catenulatum*, and *B. pseudocatenulatum*. The genomic ANIm of N4G05 was also on the borderline of the specified cutoff for species identity, with a maximum ANIm of 94.7% with *B. kashiwanohense*, followed by 94.5% with *B. catenulatum*.

The phenotypes of N4G05 and N5G01 have been described previously, including carbohydrate fermentation pattern, production of lactic acid and hydrogen peroxide, tolerance to low pH, and antibiotic susceptibility (10).

### Accession number(s).

This whole-genome shotgun project has been deposited in GenBank under the accession no. NJNQ00000000 for strain N4G05 and NJNP00000000 for strain N5G01. The versions described in this paper are NJNQ01000000 and NJNP01000000, respectively.
